# CDKN2A was a cuproptosis-related gene in regulating chemotherapy resistance by the MAGE-A family in breast cancer: based on artificial intelligence (AI)-constructed pan-cancer risk model

**DOI:** 10.18632/aging.205125

**Published:** 2023-10-19

**Authors:** Hong Wan, Xiaowei Yang, Guopeng Sang, Zhifan Ruan, Zichen Ling, Mingzhao Zhang, Chang Liu, Xiangyang Hu, Tao Guo, Juntong He, Defeng Liu, Jing Pei

**Affiliations:** 1 Department of General Surgery, The First Affiliated Hospital of Anhui Medical University, Hefei, Anhui, China; 2 Anhui Public Health Clinical Center, Hefei, Anhui, China; 3 Department of Breast Surgery, The First Affiliated Hospital of Anhui Medical University, Hefei, Anhui, China; 4 Department of Pathology, The First Affiliated Hospital of Anhui Medical University, Hefei, Anhui, China

**Keywords:** cuproptosis, CDKN2A, breast cancer, artificial intelligence (AI), model

## Abstract

Background: Before the discovery of cuproptosis, copper-loaded nanoparticle is a wildly applied strategy for enhancing the tumor-cell-killing effect of chemotherapy. Although copper(ii)-related researches are wide, details of cuproptosis-related bioprocess in pan-cancer are not clear yet now, especially for prognosis and drug sensitivity prediction yet now.

Methods: In this study, VOSviewer is used for the literature review, and R4.2.0 is used for data analysis. Public data are collected from TCGA and GEO, local breast cancer cohort is collected to verify the expression level of CDKN2A.

Results: 7036 published articles exhibited a time-dependent linear relationship (R=0.9781, p<0.0001), and breast cancer (33.4%) is the most researched topic. Cuproptosis-related-genes (CRGs)-based unsupervised clustering divides pan-cancer subgroups into four groups (CRG subgroup) with differences in prognosis and tumor immunity. 44 tumor-driver-genes (TDGs)-based prediction model of drug sensitivity and prognosis is constructed by artificial intelligence (AI). Based on TDGs and clinical features, a nomogram is (C- index: 0.7, p= 6.958e- 12) constructed to predict the prognosis of breast cancer. Importance analysis identifies CDKN2A has a pivotal role in AI modeling, whose higher expression indicates worse prognosis in breast cancer. Furthermore, inhibition of CDKN2A down-regulates decreases Snail1, Twist1, Zeb1, vimentin and MMP9, while E-cadherin is increased. Besides, inhibition of CDKN2A also decreases the expression of MEGEA4, phosphorylated STAT3, PD-L1, and caspase3, while cleaved-caspase3 is increased. Finally, we find down-regulation of CDKN2A or MAGEA inhibits cell migration and wound healing, respectively.

Conclusions: AI identified CRG subgroups in pan-cancer based on CRGs-related TDGs, and 44-gene-based AI modeling is a novel tool to identify chemotherapy sensitivity in breast cancer, in which CDKN2A/MAGEA4 pathway played the most important role.

## INTRODUCTION

Tumor metabolic reprogramming is reported as an adaptive change to the microenvironment; the “Warburg effect” is known as aerobic glycolysis, and is the most widely accepted tumor feature [1, 2]. However, aerobic glycolysis in tumors cannot replace the contribution of the mitochondrial tricarboxylic acid (TCA) cycle in tumor energy provision [3–5]. Recent studies have shown that most cancer cells don’t undergo respiration and harness the TCA cycle to support tumor growth [6]. Increasing evidence suggests that some enzymes in the TCA cycle, such as pyruvate carboxylase, are critical for the growth of primary tumors or metastases [7, 8]. Furthermore, the precisely controlled intracellular reactive oxygen species (ROS) levels in tumors during oxidative metabolism are important in promoting tumorigenesis and progression [9].

Metal ions, including Fe, Cu, and Zn, are important functional units in cellular energy metabolism [10]. Among them, Cu is involved in various metabolic activities in normal physiological processes, including mitochondrial respiration, iron transport, oxidative phosphorylation, and other processes [10]. In the mitochondrial respiratory chain, Cu is reported to be involved in the formation of the mitochondrial cytochrome oxidase complex (COX), which also named mitochondrial electron transport chain complex IV, and in energy production and maintenance of the mitochondrial electrochemical gradient [11–13]. In other words, Cu plays a key role in tumor energy metabolism, especially for tumor which one depends on mitochondrial energy metabolism (breast cancer, colon cancer, etc.). In addition, depletion of intracellular Cu plays an important role in promoting glycolysis [14]. Furthermore, it has been established that the metabolites produced during aerobic glycolysis play an important role in tumor growth, metastasis, and drug resistance [15].

Given the importance of Cu in tumor energy metabolism, including regulation of the mitochondrial respiratory chain and ROS, a “copper-targeted antitumor” model that targeted mitochondrial Cu depletion to block TCA cycle-mediated tumor energy metabolism is established to inhibit tumor growth [13, 16]. Intriguingly, Cu-overload-induced ROS imbalance is widely thought to be a novel strategy for antitumor therapy [17, 18]. Interestingly, a pivotal study defined “copper-induced cell death” as an excess of Cu in tumor cells that directly binds to the lipoylation component (DLAT) in the CTA cycle to induce an abnormal aggregation of lipoylated proteins and interfere with intracellular iron-sulfur cluster proteins, to induce cell death by proteotoxicity [19]; this process is termed cuproptosis.

Cuproptosis is a novel copper metabolism-mediated tumor-suppressive process independent of ferroptosis, which provides a novel strategy for copper-loaded nanoparticles in antitumor therapy. However, more research is warranted to uncover the roles of cuproptosis in pan-cancer, including cuproptosis-induced tumor microenvironment (TME) immune cell infiltration and energy metabolism reprogramming. Up to now, few studies have assessed the effects of cuproptosis-related genes (CRGs) in chemotherapy sensitivity prediction [20], especially for breast cancer.

This study aims to explore the relationship between cuproptosis and tumor immunity, and to construct a risk model to predict clinical outcomes and chemotherapy resistance.

## MATERIALS AND METHODS

**Figure 1:**
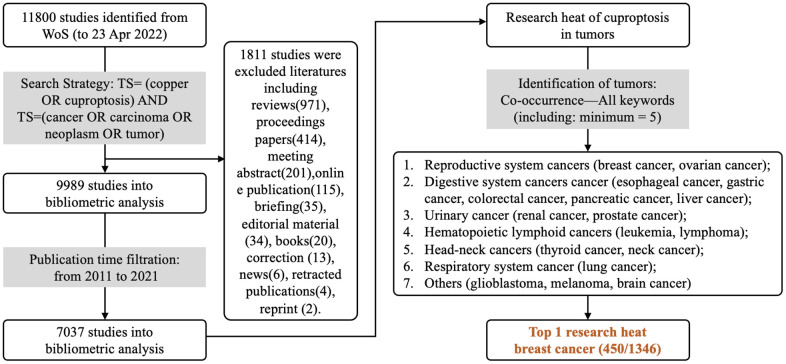
**Bibliometric analysis process.** WOS is used for researching copper(II)-related studies in tumors, using the search strategy “TS= (copper OR cuproptosis) AND TS= (cancer OR carcinoma OR neoplasm OR tumor)” for publications from 2011-2021, and only articles are collected until April 23, 2022, yielding 7037 articles.

The research process of bibliometric analysis is shown in Figure 1. The bibliometric analysis is conducted until April 23, 2022, and data analysis is performed by two researchers independently. All abbreviations are listed in Supplementary Table 1.

### Bibliometric analysis

### Data sources and search strategies

Web of Science (WOS) is used to collect copper(II)-related publications until April 23, 2022. The search strategy is as follows: TS= (copper OR cuproptosis) AND TS= (cancer OR carcinoma OR neoplasm OR tumor). 11800 pieces of literature are obtained, including articles (n=9989) and non-articles (reviews, proceeding papers, or others, n=1811) (Figure 1). After screening studies published between 2011 to 2021, 7037 studies are finally included.

### Data analysis

First, we applied VOS viewer 16.0 (Leiden University Science and Technology Research Center, Leiden, Netherlands, a software used in bibliometric analysis before [21]) software to clean the collected data and merge duplicates or synonyms through the thesaurus file. We finally collected keywords, journal names, research institution, research country, and article citations for further data analysis, yielding a total of 7036 articles. VOSviewer 16.0 is used for literature review and bibliometric analysis, amongst which keywords are used for quantifying copper(II)-related studies in different cancers. R4.2.0 is used for cox regression analysis; results are visualized in nomograms, calibration curves, and gene expression heatmaps. Data from Kaplan Meier-plotter are divided into training and internal validation groups. Data from TGCA (The Cancer Genome Atlas) are used for external validation.

The number of publications (NP) and the number of citations (NCs) are used for calculating the mean number of citations (MNCs). The relationship between NPs and time (year) is analyzed via simple linear regression in Statistical Product Service Solutions 20.0 (SPSS 20.0) and plotted by GraphPad Prism 7.0. The worldwide distribution map of the NPs is plotted in R4.2.0 (packages: *maps, ggplot*). The entry frequency threshold of the keyword is 50, and the entry frequency threshold of the journal is 20. The tumor distribution of copper(II) is calculated based on the frequency of the tumor name and the applied cell line mentioned in the keywords and plotted by VOS viewer 16.0. In addition, the keyword distribution is also plotted by VOS viewer 16.0.

### Cuproptosis in pan-cancer

### Data collection

All expression profiles and clinical data of pan-cancer (31 types of cancer: Adrenocortical Carcinoma [ACC], Bladder Urothelial Carcinoma [BLCA], Breast Invasive Carcinoma [BRCA], Cervical Squamous Cell Carcinoma and Endocervical Adenocarcinoma [CESC], Cholangiocarcinoma [CHOL], Colon Adenocarcinoma [COAD], Lymphoid Neoplasm Diffuse Large B-cell Lymphoma [DLBC], Esophageal Carcinoma [ESCA], Glioblastoma Multiforme [GBM], Head and Neck Squamous Cell Carcinoma [HNSC], Kidney Chromophobe [KICH], Kidney Renal Clear Cell Carcinoma [KIRC], Kidney Renal Papillary Cell Carcinoma [KIRP], Acute Myeloid Leukemia [LAML], Brain Lower Grade Glioma [LGG], Liver Hepatocellular Carcinoma [LIHC], Lung Adenocarcinoma [LUAD], Lung Squamous Cell Carcinoma [LUSC], Mesothelioma [MESO], Ovarian Serous Cystadenocarcinoma [OV], Pancreatic Adenocarcinoma [PAAD], Pheochromocytoma and Paraganglioma [PCPG], Prostate Adenocarcinoma [PRAD], Rectum Adenocarcinoma [READ], Sarcoma [SARC], Skin Cutaneous Melanoma [SKCM], Stomach Adenocarcinoma [STAD], Testicular Germ Cell Tumors [TGCT], Thyroid Carcinoma [THCA], Thymoma [THYM], Uterine Corpus Endometrial Carcinoma [UCEC], Uterine Carcinosarcoma [UCS], Uveal Melanoma [UVM]) are obtained from TCGA (https://portal.gdc.cancer.gov), and data cleaning is performed in R4.2.0. The normalized expression profile of the pan-cancer is collected from UCSC Xena (http://xena.ucsc.edu), and 9593 samples are finally included. Breast cancer gene expression profiles are collected from Gene Expression Omnibus (GEO: https://www.ncbi.nlm.nih.gov/geo/), in which 538 samples with follow-up data are collected from GSE20685, GSE58812, and GSE42568. Triple-negative breast cancer (TNBC) gene expression profile is collected from GSE18864, GSE58812, GSE76124, GSE83937, and GSE95700 (n=517). 16 independent GEO cohorts (GSE5460, GSE12276, GSE27830, GSE31448, GSE32646, GSE42568, GSE58984, GSE65194, GSE66305, GSE76275, GSE102484, GSE129556, GSE146558, GSE147472, GSE167213, GSE199135) are merged into a 2953-sample-constructed verification cohort. Immunohistochemistry of CDKN2A in breast cancer tissues is collected from The Human Protein Atlas (https://www.proteinatlas.org). Cuproptosis-related genes (CRGs) are collected from Todd R Golub et al.’s research (PMID: 35298263, DOI: 10.1126/science.abf0529), including CDKN2A (Cyclin-dependent Kinase Inhibitor 2A), DLAT (Dihydrolipoamide S-acetyltransferase), DLD (Dihydrolipoamide Dehydrogenase), FDX1 (Ferredoxin 1), GLS (Glutaminase), LIAS (Lipoic Acid Synthetase), LIPT1 (Lipoyltransferase 1), MTF1 (Metal-regulatory Transcription Factor 1), PDHA1 (Pyruvate Dehydrogenase α1), PDHB (Pyruvate Dehydrogenase).

### Data analysis

### Univariate cox regression and expression map

The prognosis hazard ratio of CRGs in pan-cancer is calculated by R4.2.0 using the survival packages; p-value<0.05 is statistically significant, and the distribution map of pan-cancer is plotted by the online tool in “OmicShare Tools” (https://www.omicshare.com/tools). The different expression genes are gained from Gene Expression Profiling Interactive Analysis 2 (GEPIA2, http://gepia2.cancer-pku.cn/), with the significance criteria: log2FC>1.0 and q-value<0.01. The gene heatmap is plotted in OmicShare Tools.

### Multivariate cox regression and risk model

10 CRGs are used to construct multi-gene risk model by multivariate cox regression in R4.2.0 (*coxph* function).

### IRGs-based subgroups identification

*ConsensusClusterPlus* package in R4.2.0 is used to perform consensus clustering analysis, based on the CRGs (*parameter: maxK=10, reps=50*).

### CRG-TDGs-based artificial intelligence (AI) modeling

Simply, the *limma* package is used to identify CRGs-related TDGs (48 genes are identified), followed by univariate cox regression, and finally 44 prognosis-related CRG-TDGs are identified. Then, artificial intelligence (AI) is used to construct and train the model, in which six AI functions are applied, including K-Nearest Neighbor (KNN, *kknn* package), extreme gradient boosting (XGboost, *xgboost* package), multi-logistic (*nnet* packages), support vector machine (SVM, *e1071* packages), random forest (RF, *randomForest* package), and deep learning (DL, *h2o* package). In model construction, 75% samples in cohort are selected as the training cohort randomly, and the last one is testing cohort. Gene expression is standardized to a range of “0~1” with *preProcess* function (*caret* and *tidyverse* packages). The parameters and of AI and their codes are listed in supplementary-1 (AI_code.R).

### Drug sensitivity prediction

Drug sensitivity prediction is performed with genome by the *oncoPredict* package in R4.2.0, which is used in the previous study [22, 23].

### Biological experiments

### Clinical sample collecting

50 breast cancer and 21 para-tumor slides are collected from patients who underwent surgery in the department of breast surgery of The First Affiliated Hospital of Anhui Medical University from February 2022 to December 2022. All of the above experiments are approved by the Medical Ethics Committee of The First Affiliated Hospital of Anhui Medical University. All patients with breast cancer are confirmed by at least two pathologists.

### Immunohistochemistry

Procedure of immunohistochemistry (IHC) for CDKN2A expression level are performed as previously described (PMID: 23200678 and 20571492). Simply, the work concentration of antibody against CDKN2A (GB111143, Servicebio, China) is 1:100. The protein expression level is assessed by Mean of Integrated Option Density (IOD) with Image-ProR Plus. Briefly, all of the immunohistochemical sections are photographed for three yields in the same standard, and then select Area of Interesting (AOI) and detect IOD to gain the Mean of IOD (IOD/AOI, MI). Finally, CDKN2A expression level is divided into a high and low group according to the Mean of MI.

### Statistical analysis

All data analyses are performed in R4.2.0, amongst which Pearson’s test and Wilcox rank sum test and Kruskal Wallis rank sum test are used for calculating the correlation between different genes and assessing differences for continuous variables, respectively. Univariate cox regression is performed to calculate the hazard ratio (HR) and the log-rank test is used to compare survival differences. Receiver operating characteristic (ROC) curves and the AUC value is performed by *pROC* package in R4.2.0. GO and KEGG analyzes are performed by *clusterProfiler* package in R4.2.0. *P<0.05* is considered to indicate a statistically significant difference.

### Data availability statement

The original contributions presented in the study are included in the article Supplementary Material. Further inquiries can be directed to the corresponding authors.

## RESULTS

We explore the correlation between cuproptosis and tumor immunity evasion, and further uncover the effects of cuproptosis-related genes in predicting tumor prognosis and chemotherapy sensitivity in pan-cancer.

### Overview of research status and tumor distribution of copper (II)-related studies

**Figure 2:**
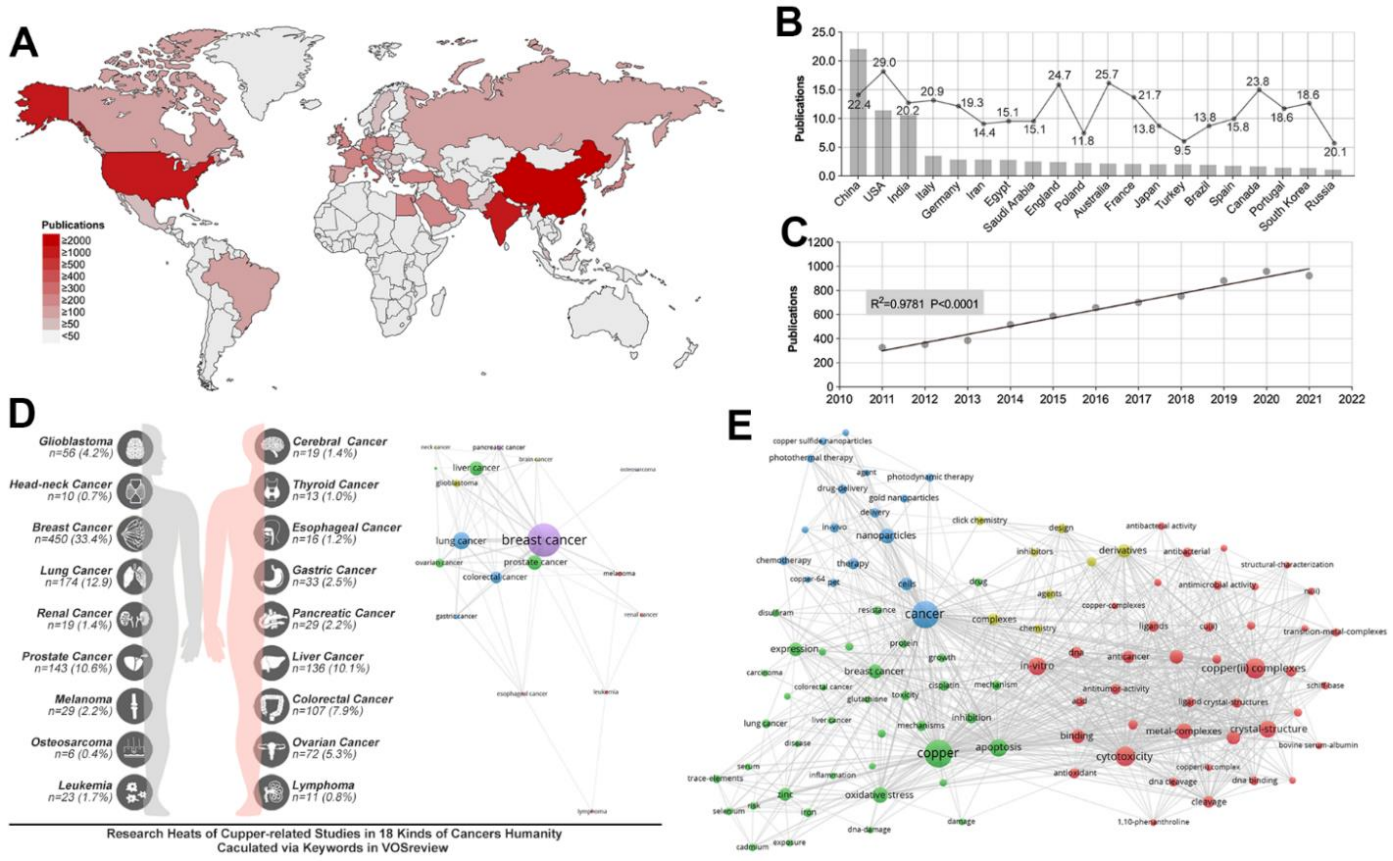
**Research status and publication trend of copper(II)-related studies.** (**A**) Analysis of the worldwide distribution of publications showed China, India and USA are the most prolific countries. (**B**) Analysis of the mean article citations in the most prolific 20 countries showed the USA made the most significant impact in copper(II)-related research. (**C**) A linear relationship is found between NPs and time (R square = 0.9781, p<0.0001). (**D**) Copper(II)-related studies distribution in pan-cancer showed breast cancer, lung cancer and prostate cancer are the top 3 research fields. (**E**) Keywords distribution.

The literature retrieval process is conducted until April 23, 2022, and the number of publications (NPs) from 2010 to 2021 is quantified, yielding 7037 articles which meet the inclusion criteria (Figure 1). We analyze the worldwide distribution of publications, which showed that CHINA (n=2209, 31.4%), USA (n=114, 16.3%), and INDIA (n=1057, 15.0%) are the most prolific countries (Figure 2A). Analysis of the mean number of citations (MNCs) shows SLOVENIA (MNCs=38.3) make the most impact in copper(II)-related research, while the USA, CHINA, and INDIA ranked 4^th^, 18^th^, and 24^th^, respectively, in contributing to copper(II)-related research (Figure 2B). In addition, the Journal of Inorganic Biochemistry held the most publications on copper(II)-related studies (Table 1). In addition, the top 10 prolific organizations are calculated, and results show that University of the Chinese Academy of Sciences (NPs= 61, MNCs=55.3) make the most impact in copper(II)-related research, and the Chinese Academy of Sciences (NPs= 211, MNCs=39.3) is the most prolific (Table 2). Besides, Duke University ranked second in making an impact in copper(II)-related research, and Aligarh Muslim University (NPs=106, MNCs=24.7) ranks second in NPs (Table 2). Annual publication analysis shows that NPs increasing in a linear model (R2 = 0.978, P<0.0001) (Figure 2C). Moreover, the frequency of copper(II)-related studies in various tumors is calculated by the frequency of tumor name and cell line in the keywords. Results show that breast cancer (n=450, 33.4%) is the most researched topic associated with copper(II), which is followed by lung (n=174, 12.9%), prostate (n=143, 10.6%), and liver (n=136, 10.1%) cancers (Figure 2D). Keyword statistics showed that “copper(II) (n=1627), cancer (n=1591), copper(II) complexes (n=883), cytotoxicity (n=830), apoptosis (n=702), *in-vitro* (n=672), crystal-structure (n=636), metal-complexes (n=536), oxidative stress (n=528), and DNA-banding (n=424)” are top 10 most researched fields (Figure 2E).

**Table 1:** The top 10 most prolific journals on copper(II)-related publication.

**Rank**	**Journal**	**NPs**	**NCs**	**MNCs**	**IF (2020)**
1	J INORG BIOCHEM	213	4862	22.8	4.155
2	DALTON T	175	5377	30.7	4.390
3	INORG CHIM ACTA	145	2051	14.1	2.545
4	J MOL STRUCT	143	1768	12.4	3.196
5	RSC ADV	136	2355	17.3	3.361
6	APPL ORGANOMET CHEM	131	1163	8.9	4.105
7	EUR J MED CHEM	111	4233	38.1	6.514
8	NEW J CHEM	99	1301	13.1	3.591
9	POLYHEDRON	92	1517	16.5	3.052
10	MOLECULES	81	839	10.4	4.411

All journals (entry frequency threshold = 20) are ranked, and the most prolific journal is J INORG BIOCHEM (NPs=213, MNCs=22.8, IF=4.155).

**Table 2:** The top 10 most prolific organizations.

**Rank**	**Organization**	**Country**	**NPs**	**NCs**	**MNCs**
1	CHINESE ACAD SCI	CHINA	211	8294	39.3
2	ALIGARH MUSLIM UNIV	INDIA	106	2622	24.7
3	GUANGXI NORMAL UNIV	CHINA	84	1537	18.3
4	KING SAUD UNIV	SAUDI ARABIA	84	1897	22.6
5	UNIV LISBON	PORTUGAL	81	1512	18.7
6	SUN YAT SEN UNIV	CHINA	70	1523	21.8
7	NANJING UNIV	CHINA	67	1297	19.4
8	UNIV CHINESE ACAD SCI	CHINA	61	3374	55.3
9	UNIV SAO PAULO	BRIZAL	60	733	12.2
10	SHANGHAI JIAO TONG UNIV	CHINA	58	1697	29.3

All organizations (entry frequency threshold = 20) are ranked, and the most prolific organization is CHINESE ACAD SCI (NPs=211, MNCs=39.3).

### Expression and prognosis hazard of CRGs in pan-cancer

**Figure 3:**
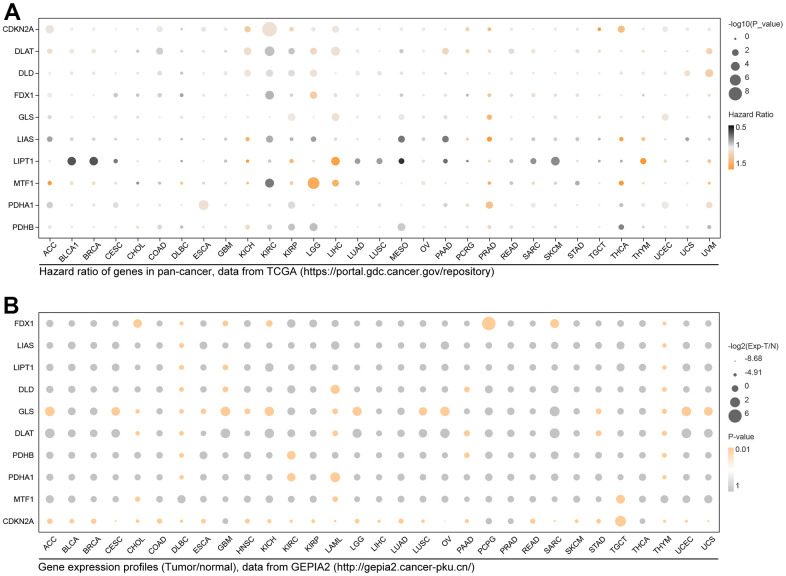
**Expression distribution and prognosis hazard of CRGs in pan-cancer.** (**A**) CDKN2A has prognostic significance in 6 types of cancer; DLAT in 10 types of cancer; DLD in 6 types of cancer; FDX1 in KIRC and LGG; GLS in 4 types of cancer; LIAS in 5 types of cancer; LIPT1 in 9 types of cancer; MTF1 in 3 types of cancer; PDHB1 in 7 types of cancer; LIAS in 5 types of cancer; PDHB in 5 types of cancer. (**B**) Differential expression of FDX1 in 7 types of cancer; LISA in DLBC and THYM; LIPT1 in DLBC, GBM, and THYM; DLD in 5 types of cancer; GLS in 16 types of cancer; DLAT in 6 types of cancer; PDHB in DLBC, KIRC, PAAD, and THYM; PDHA1 in DLBC, KIRC, LAML, and THYM; MTF1 in CHOL, LAML, and TGCT; CDKN2A in 27 types of cancer.

To learn the effects of CRCGs in cancers, we explored the expression features and prognosis hazard ratio (HR) in pan-cancer. As the results show in Figure 3A, CDKN2A has prognostic significance in 6 types of cancer (ACC, COAD, KICH, KIRC, LIHC, THCA, UCEC), and DLAT, DLD, FDX1, GLS, LIAS, LIPT1, MTF1, PDHA1 and PDHB have different prognosis significance in pan-cancer (Figure 3A). ANOVA analysis result of differential expression levels in pan-cancer performed in GEPIA2 are shown in Figure 3B, with a threshold of p<0.01 and logFC≥1.

### CRGs-based multi-gene risk-score in pan-cancer

**Figure 4:**
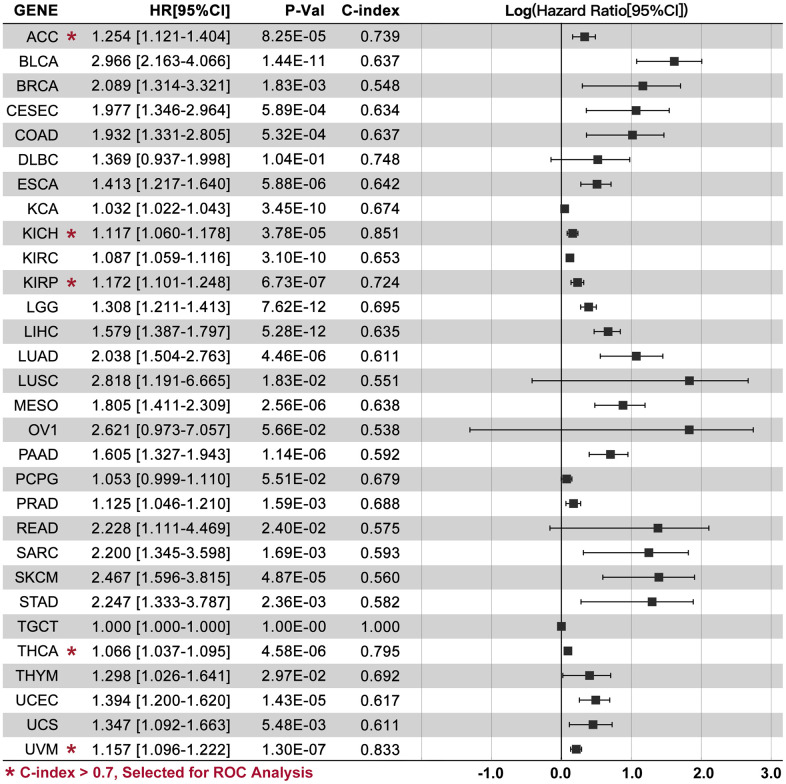
**CRCGs-based multi-gene risk-score.** TCGA data are used to construct a multi-gene risk-score model, and C-index is higher than 0.7 in ACC (HR=1.254[1.121-1.], P=8.25e-05), KICH (HR=1.117[1.060-1.178], P=3.78e-05), KIRP (HR=1.172[1.101-1.248], P=6.73e-07), THCA (HR=1.066[1.037-1.095], P=4.58e-06), and UVM (HR=1.157[1.096-1.222], P=1.30e-07).

**Figure 5:**
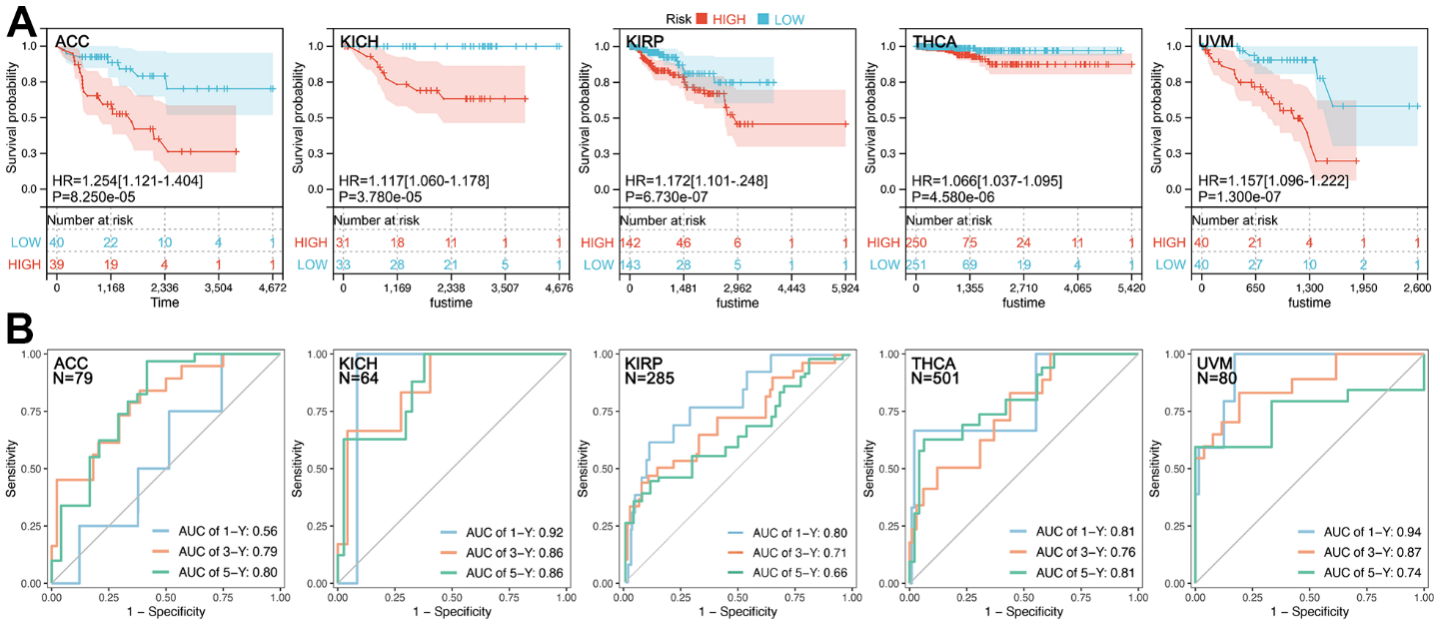
**Prognosis hazard and assessment efficiency of CRCGs-based multi-gene risk-score.** (**A**) Higher CRCGScore predicted worse prognosis in ACC (HR=1.254, P=8.250e-05), KICH (HR=1.117, P=3.780e-05), KIRP (HR=1.172, P=6.730e-07), THCA (HR=1.066, P=4.580e-06), and UVM (HR=1.157, P=1.300e-07). (**B**) ROC values for survival at 1, 3 and 5 years 0.56, 0.79, and 0.80 in ACC, 0.92, 0.86, and 0.86 in KICH, 0.80, 0.71, and 0.66 in KIRP, 0.81, 0.76, and 0.81 in THCA and 0.94, 0.87, and 0.74 in UVM, respectively.

CRGs-based Multi-gene Risk-score (CRGScore) is not significant in DLBC, OV, PCPG, and TGCT (Figure 4, P>0.05), while a relatively good performance of the CRGScore is observed in ACC (C-index=0.739, P=8.25e-05), KICH (C-index=0.851, P=3.78e-05), KIRP (C-index=0.724, P=6.73e-07), THCA (C-index=0.795, P=4.58e-06), and UVM (C-index=0.833, P=1.30e-07). Moreover, different CRGs are used to construct the risk-score model in ACC (CDKN2A, DLAT, and PDHA1), KICH (CDKN2A, DLD, GLS, LIPTA, and PDHA1), KIRP (DLAT, GLS, and PDHA1), THCA (CDKN2A, DLD, LIAS, PDHA1, and PDHB), and UVM (DLD, LIAS, MTF1, and PDHB). Kaplan-Meier (K-M) analysis shows that a higher CRGScore predicts a worse prognosis in ACC, KICH, KIRP, THCA, and UVM (Figure 5A). Besides, ROC analysis assessed the efficiency of CRCGScore in predicting prognosis. The results show that ROC values for survival at 1, 3, and 5 years are 0.56, 0.79, and 0.80 in ACC, 0.92, 0.86, and 0.86 in KICH, 0.80, 0.71, and 0.66 in KIRP, 0.81, 0.76, and 0.81 in THCA and 0.94, 0.87, and 0.74 in UVM, respectively (Figure 5B).

### Identification of CRG subgroups and features in pan-cancer

**Figure 6:**
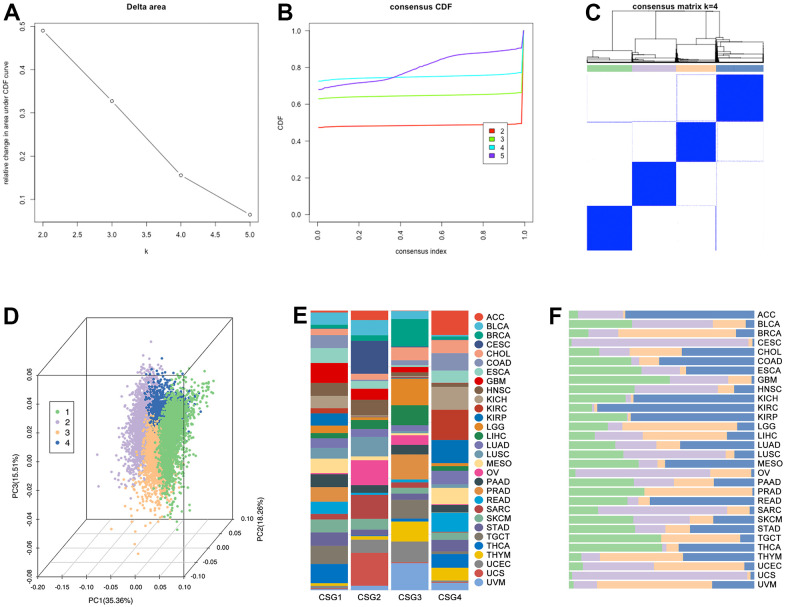
**CRG subgroups identification in pan-cancer.** Based on (**A**) delta area and (**B**) consensus CDF, clustering analysis divided pan-cancer into (**C**) four groups, which are renamed CSG1 (green), CSG2 (purple), CSG3 (orange), and CSG4 (blue). (**D**) Co-PCA analysis displayed sample disturbance amongst four CRG subgroups. (**E**) Cancer-type disturbance in the subgroup and (**F**) subgroup disturbance in single cancer type are explored.

**Figure 7:**
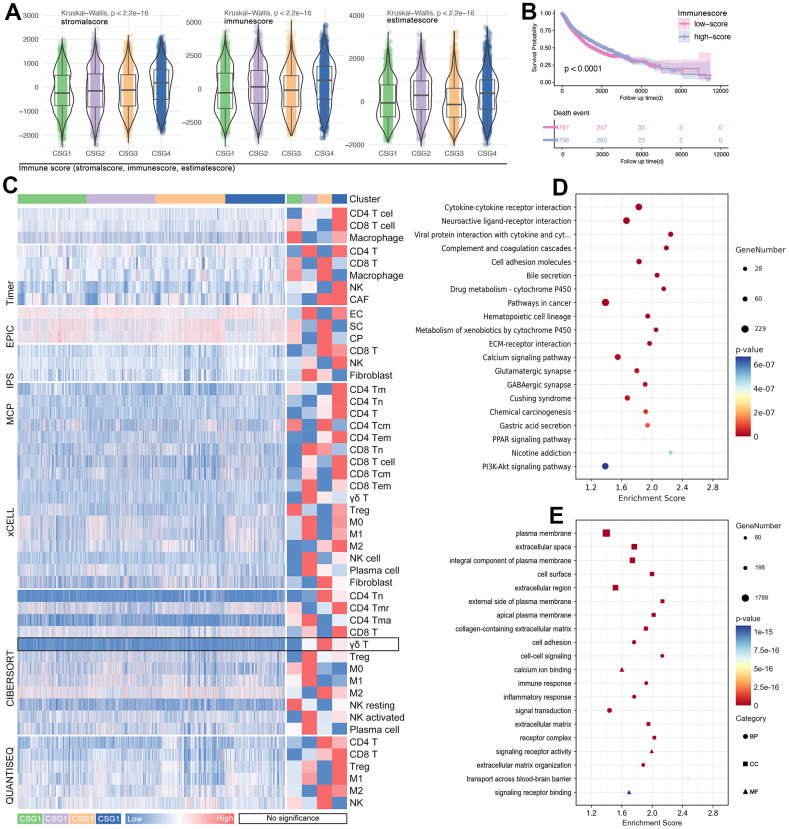
**Tumor immunity and pathway features of CRG subgroups.** (**A**) Stormalscore, immunescore, and estimatescore are predicted in R4.2.0. (**B**) K-M analysis showed the effect of immunescore on prognosis in pan-cancer. (**C**) Immune cell infiltration in CRG subgroups, including innate immunity and adjust immunity. (**D**) KEEG and (**E**) GO analysis showed CRG-related-genes-related molecular pathways.

The results display that four groups are the best division strategy (Figure 6A, 6B), and the clustering is observed obviously (Figure 6C, 6D). Besides, the proportion of pan-cancer in each subgroup is significantly different (Figure 6E). At the same time, the proportion of subgroups in different single tumor types is also different (Figure 6F). Then, features of CRG subgroups are explored. As the Figure 7A shows, CSG4 holds highest score of stromalscore, immunescore and estimatescore (p<2.2e-16, Figure 7A). Kaplan-Meier analysis shows higher immunescore with better prognosis (p<0.0001, Figure 7B). Following, immune cells infiltration in tumor tissues is explored, and the results showed that immune cells are differently infiltrate in four CRG subgroups (Figure 7C). Then, CRGs-related genes are put into KEGG and GO analysis to further unclear the roles of cuproptosis in regulating tumor immunity. As the results show, CRGs-related genes are involved in Drug Metabolism, Cytokine-cytokine Receptor Interaction, Immune Response, and Inflammatory Response, et al. (Figure 7D, 7E). Both of the above molecular pathway analyses imply that cuproptosis is related with tumor immunity.

**Figure 8:**
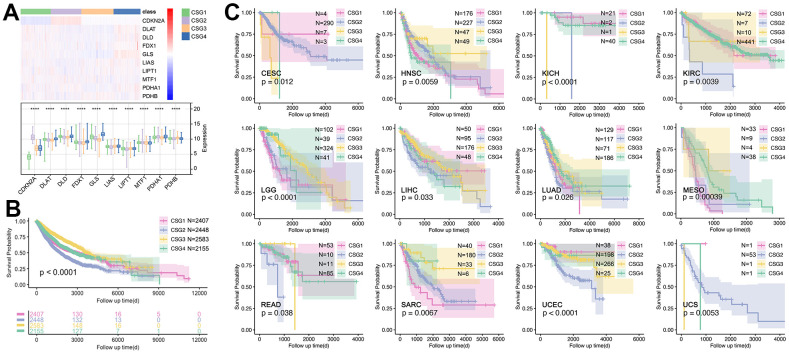
**Expression profiles and prognosis features of CRG subgroups.** (**A**) Expression profile of 10 CRGs in CRGs. (**B**) Prognosis differences of CRG subgroups. (**C**) CRG subgroup division identified the prognosis differences in single cancers, including CESC, HNSC, KICH, KIRC, LGG, LIHC, LUAD, MESO, READ, SARC, UCEC, and UCS.

Next, the prognosis differences amongst CRGs are explored. As Figure 8A shows, CRGs hold different expressions amongst CRG subgroups, especially for CDKN2A (Figure 8A). Then, we found there is a significant difference in prognosis amongst different CRG subgroups (Figure 8B) in pan-cancer, while the prognosis differences only appeared in 12 single cancer cohorts (CESC, HNSC, KICH, KIRC, LGG, LIHC, LUAD, SO, READ, SARC, UCEC, UCS) (Figure 8C).

### CRG-TDGs-based AI modeling

**Figure 9:**
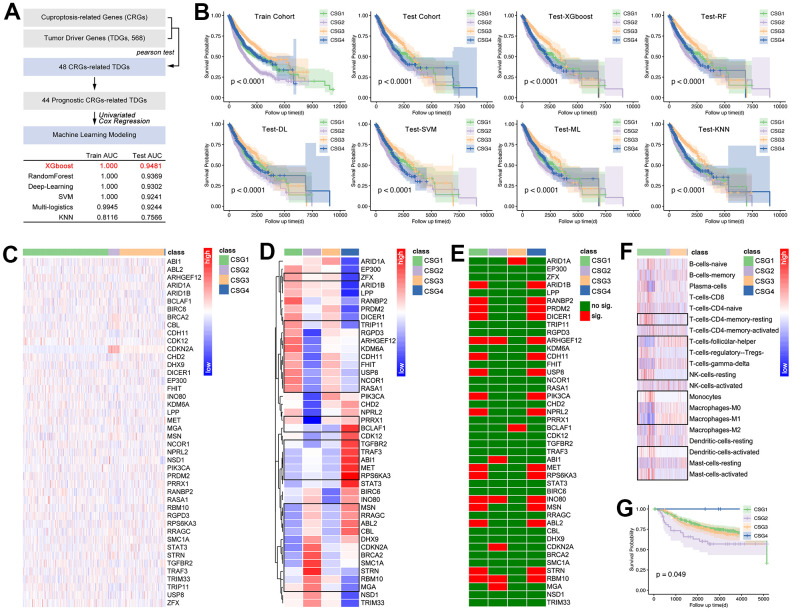
**Artificial intelligence identified CRG subgroups.** (**A**) Processing of identifying 44 target genes, and they are put into AI training. In this part, 75% of pan-cancer data are defined as a training cohort, while the last 25% is defined as a testing cohort. Six types of AI functions are performed, including XGboost, RandomForest, Deep-Learning, SVM, Multi-logistics, and KNN. (**B**) Prognosis differences amongst CRG subgroups which are identified by AI. Then, the GEO breast cancer cohort (GSE58812, GSE42568, GSE20685, n=538) is divided into four CRG subgroups by XGboost, and here displayed 44-gene expression profile (**C**) and its disturbance differences (**D**) (the representatives selected in the black box have statistical differences). Single-gene-mediated prognosis signature is explored (**E**). CIBERSORT explored immune cell infiltration features in CRG subgroups (**F**), in which the representatives selected in the black box have statistical differences. (**G**) K-M analysis showed prognosis differences amongst four CRG subgroups in breast cancer.

With the development of artificial intelligence (AI) in medicine, AI is also applied in the identification of tumor subgroups, by which a novel assessment of prognosis and drug sensitivity is constructed. Therefore, we comprehensively assess the AI in identification of CRG subgroups in pan-cancer. Firstly, 44 prognosis-related CRGs-related tumor driver genes (CRG-TDGs) are screened out, and are put into machine learning. Amongst the six types of AI, XGboost holds the best prediction efficiency, in which the training AUC is 1.000, and the testing AUC is 0.9481 (Figure 9A). In fact, the other four AI functions also perform well (Figure 9A). Following, prognosis differences amongst subgroups are explored. As the results show, prognosis difference exists in each AI-identified CRG subgroups (Figure 9B).

In order to decrease the bias caused by gene expression features in different cancers, an expression data standardization is performed, and after which the gene expression is adjusted into a range of 0 to 1. Then GEO-derived breast cancer cohort (n=538) is put into AI-driven CRG subgroup identification. Figure 9C, 9D show the expression feature of CRG-TDGs amongst CRG subgroups in breast cancer. Figure 9E shows prognosis significance of CRG-TDGs in each identified CRG subgroup in breast cancer. Following, immune cell infiltration is explored, and the result is showed in Figure 9F. Finally, Kaplan Meier (K-M) analysis showed there is a significant difference of prognosis amongst CRG subgroups (p=0.049, Figure 9G).

### CRG-TDGs-based prognosis prediction model

**Figure 10:**
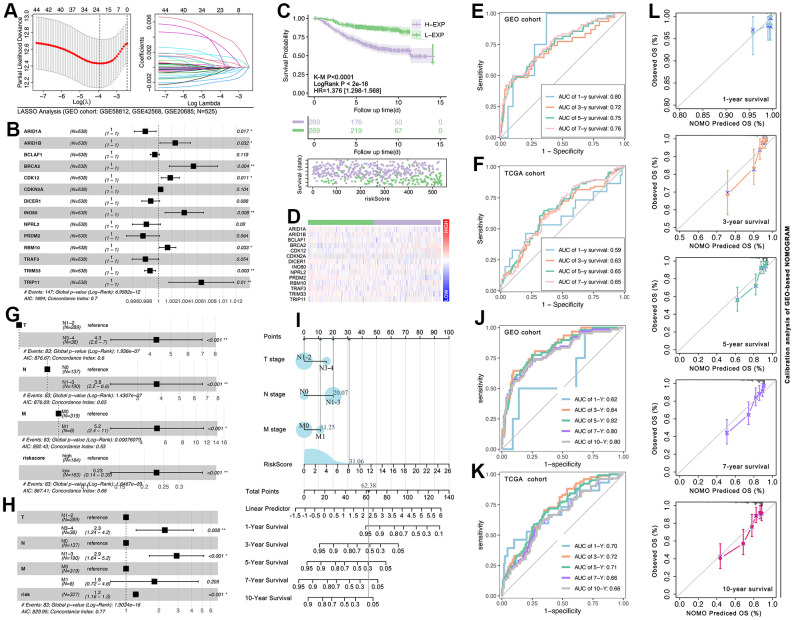
**CRG-TDGs-based nomogram construction.** (**A**) LASSO analysis. (**B**) Multivariate cox regression selected ARID1A, ARID1B, BCLAF1, BRCA2, CDK12, CDKN2A, DICER1, INO80, NPRL2, PRDM2, RBM10, TRAF3, TRIM33, and TRIP11 to construct multi-gene risk model, of which the C-index is 0.7 (log-rank p=6.9582e-12). (**C**) K-M analysis showed the prognosis difference between the high-risk group and the low-risk group, and (**D**) the gene expression profile is also explored. ROC analysis is performed to assess the prediction efficiency of the risk model in (**E**) the GEO cohort and (**F**) the TCGA cohort. (**G**) Univariate cox regression displayed that T stage, N stage, and M stage are all prognosis-related factors in the GEO breast cancer cohort, while (**H**) multivariate cox regression displayed only T stage, N stage, and risk-score are prognosis-related factors. (**I**) The multivariate cox regression model visualization display. ROC analysis is performed to assess the prediction efficiency of the nomogram in (**J**) the GEO cohort and (**K**) the TCGA cohort. (**L**) Calibration of GEO-based NOMOGRAM.

Firstly, LASSO analysis is performed to narrow the number of selected CRG-TDGs (Figure 10A). Then multivariate cox regression is performed to construct a multi-genes risk model, and the results display that the C-index of the risk model is 0.7 (p=6.95832e-12, Figure 10B). K-M and univariate cox regression analysis show that risk model identifies hierarchical prognosis risk (HR=1.376[1.298-1.568], log-rank p<2e-16, K-M p<0.0001, Figure 10C). Gene expression feature is also displayed (Figure 10D). In order to assess the prediction efficiency of risk model, receiver operating characteristic curve (ROC) is performed, and results show that the AUC value of 1-year survival, 3-year survival, 5-year survival, and 7-year survival is 0.80, 0.72, 0.75, and 0.76 in GEO cohort, while it is 0.59, 0.63, 0.65 and 0.65 in TCGA cohort (Figure 10E, 10F). To further develop the nomogram, clinical characteristics are put into analysis. As Figure 10G shows, the N stage, T stage, and M stage are all risk factors in breast cancer (p<0.001, Figure 10G). Therefore, the N stage, T stage, M stage, and risk-score are selected to construct a new nomogram (C-index=0.77, p=1.9034e-16, Figure 10H), which is displayed in Figure 10I. And ROC analysis shows that the AUC value of 1-year survival, 3-year survival, 5-year survival, 7-year survival, and 10-year survival is 0.62, 0.84, 0.82, 0.80, and 0.80 in the GEO cohort, while it is 0.70, 0.72, 0.71, 0.66 and 0.66 in the TCGA cohort (Figure 10J, 10K). Finally, calibration analysis is performed, and the results show that GEO-based NOMOGRAM has appreciable efficiency in predicting breast cancer prognosis (Figure 10L).

### CRG-TDGs-based AI model predicted drug sensitivity

**Figure 11:**
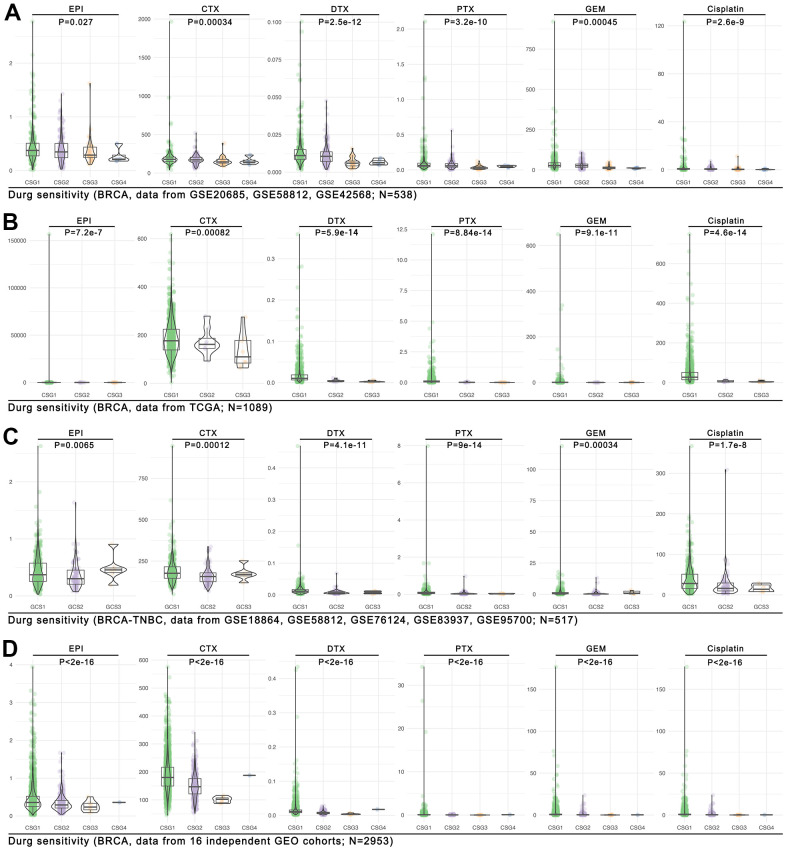
**Drug sensitivity prediction in CRG subgroups.** The OncoPredict function in R4.2.0 is performed to predict drug scores in four cohorts, and they are the (**A**) GEO breast cancer cohort (combined with GSE20685, GSE58812, GSE42568; n=538), (**B**) the TCGA breast cancer cohort (n=1089), (**C**) the GEO TNBC cohort (combined with GSE18864, GSE58812, GSE76124, GSE83937, GSE95700; n=517), and (**D**) the large GEO breast cancer cohort (combined with 16 independent cohorts; n=2953).

To our knowledge, tumor immune cell infiltration is reported to be involved in tumor drug therapy sensitivity. Here, we find CRG subgroup division identified different tumor immunizations, including NK cell infiltration, T cell infiltration, macrophage cell infiltration, et al. (Figure 9F). Therefore, the CRG subgroup division may be linked to drug sensitivity. We use the OncoPredict package in R4.2.0 to assess the drug score in each single breast cancer sample. As the Figure 11A shows, the trend of different drugs score is almost similar in the GEO breast cancer cohort (n=538, p<0.05, Figure 11A). In the TCGA breast cancer cohort (n=1089), the same trend of the drug score is observed (p<0.001, Figure 11B), while in the TNBC cohort (from GEO, n=517), we find a same trend of the drug score in DTX, PTX, GEM, and Cisplatin (p<0.001, Figure 11C). In the following further verification cohort (combined with 16 independent GEO breast cancer cohorts, n=2953), we find the trend of the drug score is also similar (p<2e-16, Figure 11D).

### CDKN2A implies malignant subtypes and regulates drug sensitivity in breast cancer

**Figure 12:**
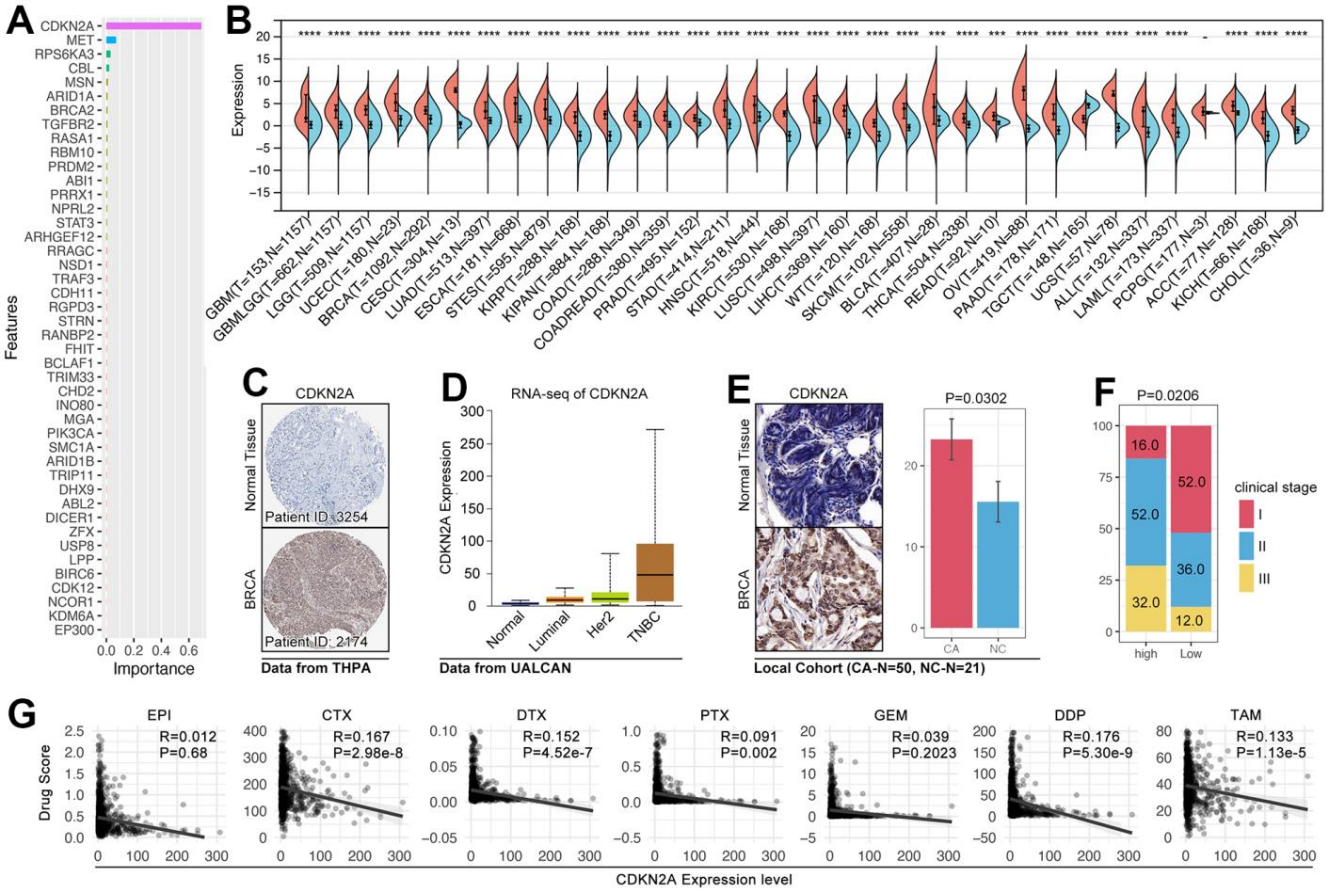
**The roles of CDKN2A in oncogenesis and drug sensitivity.** (**A**) Importance analysis displayed CDKN2A ranked top 1 in XGboost-mediated CRG subgroup identification. (**B**) The expression level of CDKN2A in pan-cancer. (**C**) IHC of CDKN2A in the breast cancer tissues and adjacent tissues (THPA: https://www.proteinatlas.org). (**D**) The mRNA expression level of CDKN2A in subtypes of breast cancer (UALCAN: http://ualcan.path.uab.edu/tutorial.html). (**E**) IHC of CDKN2A from local breast cancer. (**F**) The relationship between CDKN2A and clinical stage. (**G**) The relationship between drug score and the expression of CDKN2A.

In order to identify the pivotal genes in regulating CRG subgroup division and tumor-immunity-mediated drug sensitivity change, an importance analysis is performed, and it shows CDKN2A is the most important gene (Figure 12A). As previous studies reported, CDKN2A-mediated molecular subtypes identified drug sensitivity differences in afatinib, erlotinib, and lapatinib, and the higher expression of CDKN2A implied worse outcomes and molecular subtypes in breast cancer. In fact, CDKN2A overexpressed in 33 of 34 types of cancers, including breast cancer (Figure 12B). In order to further verify this phenotype, we collected IHC data from THPA (The Human Protein Atlas, https://www.proteinatlas.org), and the results show that CDKN2A is higher expressed in breast cancer tissues (Figure 12C). In metastasis breast cancer cohort, we find CDKN2A also overexpressed in breast cancer tissues, especially in malignant subtypes (TNBC) (Figure 12D). Collectively, in local breast cancer cohort, we found CDKN2A also overexpress in breast cancer tissues (Figure 12E and Table 3). Meantime, patients with the higher expression level of CDKN2A accompanied with worse clinical stage (p= 0.0206, Table 3). However, although above results implied CDKN2A is an oncogene in breast cancer, the expression level of CDKN2A is negatively related with drug score (CTX, DTX, PTX, DDP, and TAM) (Figure 12G).

**Table 3:** The correlation between CDKN2A expression and clinical characteristics.

**Item**		**CDKN2A Expression Level**		**P value**
		**High (n=25)**	**Low (n=25)**	
IOD		23.26±17.84	15.56±11.47	0.0302*
Clinical Stage	I	4	13	0.0206*
	II	13	9	
	III	8	3	

Samples are grouped into high-expression and low-expression group by the level of CDKN2A.

### CDNK2A mutation-mediated up-regulation of MAGEA families lead to drug resistance

**Figure 13:**
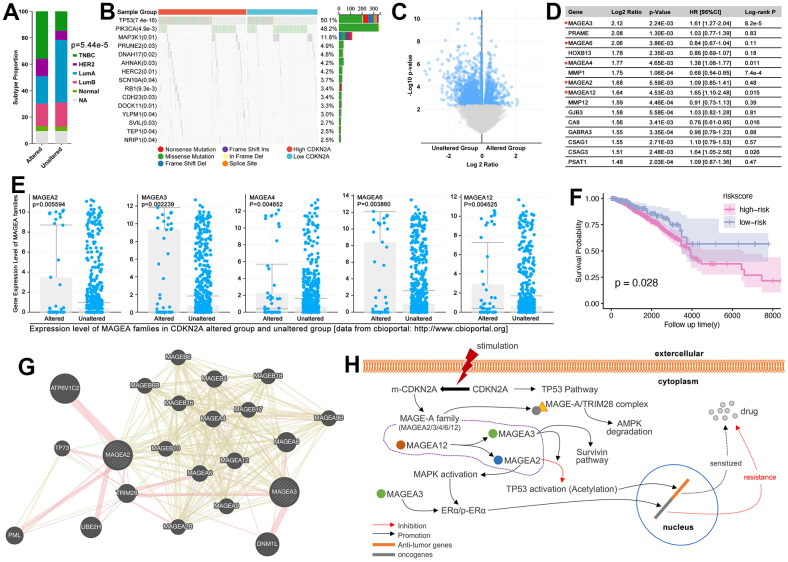
**CDKN2A-mutation/MAGE-A regulated oncogenesis and drug resistance.** (**A**) Subtype disturbance of breast cancer in CDKN2A altered group and unaltered group. (**B**) Differences of genomic mutation status between the high and low expression of CDKN2A. (**C**) Volcano plot of gene disturbance in CDKN2A altered and unaltered groups. (**D**) The top 15 different genes, amongst which MAGEA3, MAGEA4, MAGEA12, and CSAG3 are risk factors in breast cancer, while MMP1 and CA9 are protective factors in breast cancer. (**E**) The expression of the MAGE-A family in CDKN2A altered and unaltered groups. (**F**) K-M analysis of MAGE-A family-constructed multi-gene risk model. (**G**) Molecular network of MAGE-A family. (**H**) CDKN2A-mutation/MAGE-A family pathway on oncogenesis and drug resistance.

To our knowledge, CDKN2A is a cell cycle inhibitor, and it participated P53 pathway to regulate cell apoptosis and drug sensitivity [24]. However, as an anti-tumor factor, CDKN2A is up-regulated in various types of cancers (31 types of cancers, Figure 12B). As previous studies show, a high expression level of CDKN2A accompanied by the high level of methylation of itself, and the latter one is related with the high level of DNA hypermethylation, which promoted initiation of oncogenesis [25]. Except for epigenetic changes-mediated oncogenesis of CDKN2A, CDKN2A mutation-mediated drug resistance is also needed to be explored in oncogenesis and drug resistance. As the Figure 13A shows, the alteration of CDKN2A implies malignant type (TNBC) of breast cancer, while the unalteration of CDKN2A implies mild subtype (LumA) of breast cancer (p= 5.44e-5, Figure 13A). To further explore the mechanism of CDKN2A-mediated oncogenesis, we explore the expression level of CDKN2A in regulating genomic alteration. As results showed, the higher mutation level of P53 appears in the CDKN2A overexpression group (p=7.4e-16, Figure 13B). Following, we explore gene expression features in the CDKN2A mutation group. As the results showed, among the top ten genes with the most obvious expression change, the MAGE-A family gets our attention. MAGEA2, MAGEA3, MAGEA4, MAGEA6, and MAGEA12 are up-regulated in the CDKN2A alteration group, amongst which MAGEA3 (HR=1.61[1.27-2.04], p=8.2e-5), MAGEA4 (HR=1.38[1.08-1.77], p=0.011), and MAGEA12 (HR=1.65[1.10-2.48], p=0.015) are accompanied by worse prognosis in breast cancer (Figure 13C–13E). Then, MAGEA2, MAGEA3, MAGEA4, MAGEA6, and MAGEA12 are used to construct a multi-gene risk model, which shows higher risk is accompanied by worse prognosis (p=0.028, Figure 13F).

In order to uncover the mechanisms of MAGE-A family-mediated drug resistance in the CDKN2A pathway, we make a literature review firstly. To our knowledge, CDKN2A participate in P53-mediated cellular cycle regulation in the normal status of CDKN2A, while its mutation is accompanied by dysfunction of anti-tumor pathways. CDKN2A-mutation un-regulates MAGE-A family, such as MAGEA2/3/4/6/12 (Figure 13H). MAGEA12 is reported to promote the expression of MAGEA2 and MAGEA3; The higher expression of MAGEA3 is reported to be accompanied by up-regulated expression and activity of ERα, which further leads tamoxifen resistance in breast cancer. And MAGEA3 is reported to up-regulate the expression of survivin in P53-dependent and independent ways, and the latter one induced chemotherapy resistance, endocrinal therapy failure, and her2-target therapy tolerance in breast cancer [26–28]. Besides, MAGEA3 promotes MAGEA2-mediated P53 inactivation (deacetylation of P53). MAGEA2 is another important regulator in CDKN2A-mutation-mediated drug resistance. MAGEA2 is reported to promote phosphorylated activation of MAPK, which is the upstream of ERα to activate it. What more, the MAGE-A family is reported to interact with TRIM28 to initiate ubiquitous degradation of AMPK, followed by the progression of oncogenesis.

### Inhibition of CDKN2A/MAGEA4 blocks EMT and immunity escape

**Figure 14:**
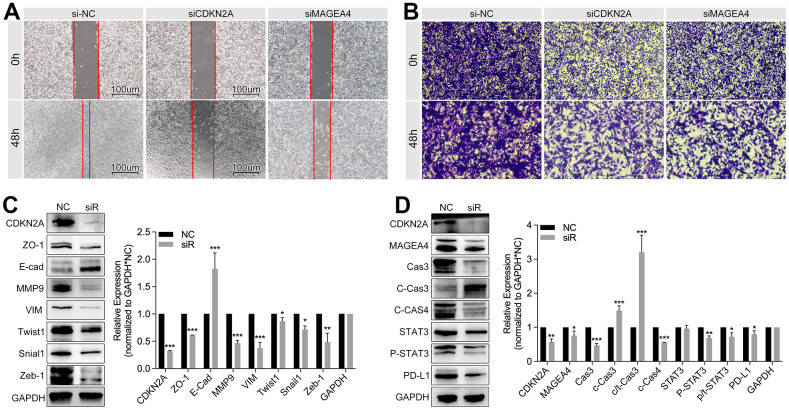
**Inhibition of CDKN2A weakens tumor metastasis and immune escape in breast cancer.** (**A**) Wound healing assay and (**B**) transwell assay show down-regulation of CDKN2A or MEGEA4 inhibits the cell migration ability. (**C**) WB detection shows down-regulation of CDKN2A decreases the expression level of ZO1, MMP9, VIM, Twist1, Snail1, and Zeb1, while E-cad is up-regulated. (**D**) Down-regulation of CDKN2A decreases the expression of MAGEA, Cas3, c-cas4, p-STAT3 and PD-L1, while c-cas3 is up-regulated.

In order to verify the regulation effects of CDKN2A/MAGEA4 in breast cancer progression, *in vitro* experiments are performed. As Figure 14A, 14B show, down-regulation of CDKN2A or MAGEA4 inhibits cell migration in wound healing assay and transwell assay. To further uncover the potential molecular mechanisms, small interfere RNA (siRNA) is used to decrease the expression of CDKN2A in TNBC cell lines. As the Figure 14C shows, down-regulation of CDKN2A decreases the expression of EMT markers, such as Snail1, Zeb1, Twist1 and MMP9, while E-cad is up-regulated. In Figure 14D, down-regulation of CDKN2A decreases the expression of phosphorylated STAT3, MAGEA4, Caspase3 and PD-L1, while cleaved-csapase3 is increased.

## DISCUSSION

Copper chelation as targeted therapy has been considered an antitumor therapy before the discovery of cuproptosis. However, no consensus has been achieved on the underlying mechanisms. On the one hand, it has been proposed that this approach leads to the depletion of intramitochondrial copper, given the pivotal role of copper in the construction of PDH in the TCA cycle. On the other hand, this approach may be conducive to intracellular copper overload, given the well-documented role of copper in producing ROS. A bibliometric analysis of copper(II)-related research found that copper is widely applied in anti-tumoral nanoparticles, including copper-containing compounds and complexes capable of binding copper. With mounting studies gradually uncovering the role of copper-induced cell death, simple intracellular overload of free copper (II) has emerged as a new antitumor strategy, which is verified by Todd R Golub et al.’s research using CuCl2.

Since Todd R Golub et al.’s research is published, the copper-induced injury is defined as a novel form of metal ions-induced mitochondrial cell death, independent of ferroptosis, which is hence named cuproptosis. During mitochondrial respiration, inactive pyruvate dehydrogenase complex (PDH) subunits (DLAT, GCSH, DBT, and DLST) are lipoylated by lipoylation enzymes LIAS, LIPT1/2, and DLD to transit to active PDH, and promote the TCA cycle. With the overloading of intracellular copper, lipoylation enzymes are inactivated by direct binding of copper, which results in aggregation-induced inactivity of lipoylation protein and destabilization of iron-sulfur cluster proteins. Finally, toxicity overload leads to cell death [19]. In the above study, FDX1, LIAS, LIPT1, DLD, DLAT, PDHA1, PDHB, MTF1, GLS, and CDKN2A are identified as the core genes to regulate cuproptosis by genome-wide CRISPR-Cas9 loss-of-function screen, amongst which FDX1 is the pivotal up-stream regulator of lipoylation enzymes, and determined cell fate upon intracellular copper overload [19]. Based on the above research, cuproptosis showed the importance of copper metabolism tumor development and supplied a novel strategy for antitumor therapy.

Recently, tumor immunity is developing into a pivotal research branch in the antitumor field. To our acknowledgment, copper also played a role in tumor immunity. Florida Voli et al.’s research showed that the increase of intracellular free copper(II) promoted phosphorylation of STAT3 and EGFR, which further maintained the expression level of PD-L1; Besides, tumor-infiltrating CD8^+^ T and NK cells are decreased by adding exogenous copper [29]. In Li X et al.’s study, increased intracellular copper(II) stabilized XIAP (E-3 ligase), and further enhanced IL17-mediated inhibition of apoptosis by activating the NF-κB pathway. Besides, copper-induced persistent inflammation promoted colon tumorigenesis [30]. Simply, it implied that copper is a regulator of tumor immunity evasion. Cuproptosis is a manifestation of copper metabolism, and cuproptosis-related proteins can regulate intracellular copper levels. Therefore, previous studies also concentrated on this problem. Qiang song et al. used CRGs to construct the multi-gene risk model, and the risk score identified T cells and dendritic cell infiltration differences in bladder cancer [31]. In fact, CRGs-based multi-gene risk models are constructed in multiple cancers, such as glioma, lung cancer, osteosarcoma, colorectal cancer, etc., and all of those studies displayed that cuproptosis is closely related to tumor immunity [32–35]. Here, we divided pan-cancer into four subtypes by CRGs-based clustering function in R4.2.0, and with the help of CIBERSORT in predicting immune cell infiltration, we found the differences of stromalscore, immunescore, and estimatescore amongst subtypes in pan-cancer (Figure 7A). In addition, the immune cell infiltration ratio displayed differences amongst CRG subgroups (Figure 7C), and enrichment analysis also showed that CRGs-related genes are involved in immunity-related molecular pathways, such as Cytokine-cytokine Receptor Interaction, Immune Response, and Inflammatory response (Figure 7D, 7E).

Although CRGs-based hierarchical risk implied different sensitivity of tumor immunity therapy, the CRGs-based risk model is not given much attention in predicting chemotherapy sensitivity, especially AI-based drug sensitivity subtype identification. With the construction of the Genomics of Drug Sensitivity in Cancer (GDSC) platform and the tumor immune score (CIBERSORT, etc.) algorithm, genome-based drug sensitivity and immunotherapy sensitivity prediction are emerging. A model based on 9 non-coding RNAs associated with epithelial-mesenchymal transition (EMT) can predict the prognosis, survival, immune score, and drug sensitivity (XELOX) of colorectal cancer [36]. In another study, Hang Zheng et al. screened the gene set of colon cancer-related tumor fibroblasts by analyzing single-cell sequencing data and constructed the prognosis model and risk grouping by LASSO analysis. The results showed that there are significant differences in prognosis, survival, and drug sensitivity between different gene expression characteristic groups (capecitabine, pazopanib, gemcitabine, sunitinib, etc.) [37]. Here, based on AI and drug sensitivity prediction, we used CRGs-related tumor driver genes (CRG-TDGs) to construct a chemotherapy sensitivity prediction model, amongst which XGboost showed the best performance (training AUC=1.000, Testing AUC=0.9481, Figure 9A). Following, we found drug sensitivity ranking is CSG1<CSG2<CSR3<CSG4, and this phenotype is verified in four different cohorts (n=5097) (Figure 11A–11D). To our knowledge, this study provided hitherto undocumented evidence of cuproptosis-based AI identifying drug sensitivity stratification in breast cancer.

To further uncover the mechanism of CRGs in regulating drug sensitivity and prognosis, we performed an importance analysis, which showed that CDKN2A is the key gene in CRG subtype identification (Figure 12A). As the previous studies reported, CDKN2A is an unstable gene and appeared alteration in various types of cancer, including pancreatic cancer, esophageal cancer, head and neck cancer, melanoma, bladder cancer, glioma, lung cancers, and etc. al. [38]. And the epigenetic (DNA methylation) or genetic (mutation) changes of CDKN2A lead to the initiation of ovarian cancer and melanoma [39, 40]. What more, CDKN2A alteration is reported to be related to immunity therapy resistance and small molecular target drug resistance in lung cancer, melanoma, and breast cancer [25, 41, 42]. However, the mechanism of CDKN2A-mutation-mediated chemotherapy resistance is still not clear. Here, we found MAGE-A family is abnormally up-regulated in the CDKN2A alteration group (Figure 13C–13E). To our knowledge, the MAGE-A family is an oncogene in various cancers, and it is verified to decrease the phosphorylated activity of P53 to promote tumor progression and drug resistance [43–45]. In breast cancer, it is verified that overexpression of MAGEA2 and MAGEA3 enhanced the activity of ERα, by which it leads to tamoxifen resistance [46]. In addition, the MAGE-A family enhanced the activity of MAPK and Survivin, both of which are wildly reported to promote drug resistance and cancer progression in breast cancer [26–28, 47–50]. Therefore, it implied that expression change of the MAGE-A family is the result of CDNK2A mutation, and it also is a novel mechanism of CDNK2A-mutation-mediated drug sensitivity in breast cancer.

Although, the detail of CDKN2A-mutation-mediated up-regulation of the MAGE-A family is not clear, and more work is necessary to uncover the mechanisms.

## CONCLUSIONS

This study explored the roles of cuproptosis-related genes in tumor prognosis and tumor immunity, and some interesting phenotypes are observed: 1. Cuproptosis-related genes identified subtypes of pan-cancer, and it implied different clinical outcomes; 2. Artificial intelligence constructed an efficient model to identify drug sensitivity subtypes in breast cancer; 3. CDKN2A has a pivotal role in subtype identification, and CDKN2A-mutation-mediated malignant subtype and drug resistance are related to the up-regulation of the MAGE-A family. However, further work is still needed to totally uncover the mechanisms of CDKN2A-mutation-mediated up-regulation of the MAGE-A family.

## Supplementary Material

Supplementary Table 1
