# Supplementary material for: CDKN2A was a cuproptosis-related gene in regulating chemotherapy resistance by the MAGE-A family in breast cancer: based on artificial intelligence (AI)-constructed pan-cancer risk model
Source: Aging (Albany NY). 2023 Oct 19;15(20):11244–67. doi: 10.18632/aging.205125 (PMC10637804; doi:10.18632/aging.205125)
Supplement: Supplementary Table 1 [file aging-15-205125-s001.pdf]

SUPPLEMENTARY TABLE

Supplementary Table 1. The abbreviation list.

Total names	Abbreviation
Adrenocortical Carcinoma	ACC
Bladder Urothelial Carcinoma	BLCA
Breast Invasive Carcinoma	BRCA
Cervical Squamous Cell Carcinoma and Endocervical Adenocarcinoma	CESC
Cholangiocarcinoma	CHOL
Colon Adenocarcinoma	COAD
Lymphoid Neoplasm Diffuse Large B-cell Lymphoma	DLBC
Esophageal Carcinoma	ESCA
Glioblastoma Multiforme	GBM
Head and Neck Squamous Cell Carcinoma	HNSC
Kidney Chromophobe	KICH
Kidney Renal Clear Cell Carcinoma	KIRC
Kidney Renal Papillary Cell Carcinoma	KIRP
Acute Myeloid Leukemia	LAML
Brain Lower Grade Glioma	LGG
Liver Hepatocellular Carcinoma	LIHC
Lung Adenocarcinoma	LUAD
Lung Squamous Cell Carcinoma	LUSC
Mesothelioma	MESO
Ovarian Serous Cystadenocarcinoma	OV
Pancreatic Adenocarcinoma	PAAD
Pheochromocytoma and Paraganglioma	PCPG
Prostate Adenocarcinoma	PRAD
Rectum Adenocarcinoma	READ
Sarcoma	SARC
Skin Cutaneous Melanoma	SKCM
Stomach Adenocarcinoma	STAD
Testicular Germ Cell Tumors	TGCT
Thyroid Carcinoma	THCA
Uterine Corpus Endometrial Carcinoma	UCEC
Uterine Carcinosarcoma	UCS
Uveal Melanoma	UVM
Cuproptosis-based subgroup	CSG
Artificial intelligence	AI
Least Absolute Shrinkage and Selection Operator	LASSO
Receptor operation curve	ROC
The Cancer Genome Atlas	TCGA
Gene Expression Omnibus	GEO
The University Alabama at Birmingham Cancer data analysis Portal	UALCAN
The Human Protein Atlas	THPA
Immunohistochemical staining	IHC
Matrix metalloproteinase 9	MMP9
vimentin	VIM
E-cadherin	E-cad
Support Vector Machine	SVM
Extreme Gradient Boosting	XGboost

Deep learning	DL
Simple sample Gene Set Enrichment Analysis	ssGSEA
Alternatively activated macrophages	M2
Classical activated macrophages	M1
CD4 T memory	CD4 Tm
CD4 T naive	CD4 Tn
CD4 T central memory	CD4 Tcm
CD4 T effective memory	CD4 Tem
CD4 T central memory	CD4 Tcm
CD4 T effective memory	CD4 Tem
